# Breastfeeding, Gestational Diabetes Mellitus, Size at Birth and Overweight/Obesity in Early Childhood

**DOI:** 10.3390/nu16091351

**Published:** 2024-04-30

**Authors:** Ye Huang, Libi Zhang, Diliyaer Ainiwan, Xialidan Alifu, Haoyue Cheng, Yiwen Qiu, Haibo Zhou, Hui Liu, Yunxian Yu

**Affiliations:** 1 Department of Public Health and Department of Anesthesiology, Second Affiliated Hospital of Zhejiang University School of Medicine, Hangzhou 310009, China; 22218854@zju.edu.cn (Y.H.); 22218857@zju.edu.cn (L.Z.); 22218236@zju.edu.cn (D.A.); 3130100017@zju.edu.cn (X.A.); 3150101365@zju.edu.cn (H.C.); yiwenqiu@zju.edu.cn (Y.Q.); 11918158@zju.edu.cn (H.Z.); 2 Department of Epidemiology & Health Statistics, School of Public Health, School of Medicine, Zhejiang University, Hangzhou 310058, China; 3 Sir Run Run Shaw Hospital, School of Medicine, Zhejiang University, Hangzhou 310016, China; lhui2010@zju.edu.cn

**Keywords:** breastfeeding, gestational diabetes mellitus, size at birth, overweight/obesity, early childhood

## Abstract

**Background:** Breastfeeding appears to reduce the risk of childhood overweight/obesity. However, it remains unclear whether this protective effect persists among high-risk populations. This study aims to investigate the association of breastfeeding with the risk of overweight/obesity in early childhood and whether this association is altered by gestational diabetes mellitus (GDM) or size at birth. **Methods:** Feeding practices during the first 12 months of age and weight and length at 12–36 months of age were collected. Full breastfeeding includes exclusive and predominant breastfeeding. Children with body mass index (BMI) values greater than 1 standard deviation from the mean of sex- and age-specific BMI were classified as overweight/obese. Multiple generalized estimating equations models were applied to analyze the associations of full breastfeeding duration with overweight/obesity risk. **Results:** Among all participants (*n* = 9329), infants with a longer full-breastfeeding duration had a reduced risk of overweight/obesity in early childhood compared with those breastfed for less than one month. Infants exposed to GDM and those born large for gestational age (LGA) had a higher risk of overweight/obesity in early childhood. Among infants of mothers with GDM (*n* = 1748), infants with full breastfeeding for greater than 6 months (aOR: 0.58; 95% CI: 0.44, 0.78) showed a decreased risk of overweight/obesity in early childhood compared with those breastfed for less than one month. Among LGA infants (*n* = 1279), infants with full breastfeeding for 3–5 months (aOR: 0.66; 95% CI: 0.57, 0.76) and greater than 6 months (aOR: 0.70; 95% CI: 0.56, 0.88) showed a decreased risk of overweight/obesity in early childhood. Similar results were observed among LGA infants of mothers with GDM. **Conclusions:** Initiating and prolonging breastfeeding would reduce the risk of overweight/obesity in early childhood, and LGA infants and infants born to mothers with GDM would experience greater benefits.

## 1. Introduction

The increasing prevalence of childhood overweight and obesity has become a pressing global public health issue, particularly in China [1,2]. The development of obesity is influenced by complex factors, including lifestyle habits, dietary patterns, and genetic predispositions [2,3]. Among these factors, the infancy stage has been identified as a critical and sensitive time window for future health [4]. Consequently, understanding the impact of infant nutrition and implementing targeted interventions has gradually become a focal point in research. Some previous studies have suggested that breastfeeding may have an impact on later health. For example, infants breastfed for longer duration have been shown to exhibit different growth trajectories [5] and neurological developments [6] compared with those breastfed for shorter periods or those fed with infant formula milk. Additionally, several studies have indicated that breastfeeding may have a protective effect against the development of obesity [7,8,9].

However, some research indicates that breast milk from mothers with hyperglycemia may alter metabolic hormone concentrations [10], which could potentially affect energy metabolism and influence the correlation between breastfeeding and offspring growth. Currently, several observational studies evaluating the association of breastfeeding with childhood overweight/obesity among women with gestational diabetes mellitus (GDM) have yielded mixed results [11,12,13,14]. For example, Gunderson et al. found that high-intensity breastfeeding during the first year was associated with reduced weight gain among offspring of mothers with GDM [12]. Conversely, another study reported that greater breastmilk intake was associated with greater weight and body mass index (BMI) gains among infants born to mothers with GDM [14]. Thus, in situations where breastfeeding is highly recommended, it is imperative to further explore the impact of breastfeeding on later overweight/obesity among offspring of mothers with GDM. In addition, size at birth, particularly being large for gestational age (LGA), was demonstrated to be closely linked to the onset of childhood obesity [15,16]. However, limited research exists that evaluates whether size at birth could alter the effect of breastfeeding on overweight/obesity.

As an in-depth supplement to existing literature, this study aims to investigate the correlation between breastfeeding and being overweight/obese in early childhood and to explore whether this association would be altered by GDM status or size at birth. By doing this, this research endeavors to provide a more comprehensive understanding of the impact of breastfeeding on offspring overweight/obesity.

## 2. Method

### 2.1. Study Design and Data Source

This is a population-based retrospective cohort study. Data were extracted from the comprehensive electronic medical record system (EMRS) of Zhoushan Maternal and Child Care Hospital, Zhejiang Province, China from 2012 to 2021. The EMRS was a municipal system established in 2001 and included information on the health care of all pregnant women and newborns in Zhoushan city after 2011 [17,18]. Briefly, according to the guidelines of Zhoushan Maternal and Child Care Hospital, the information on pregnant women was recorded in the EMRS at each regular antenatal visit, during delivery, and postpartum. Meanwhile, the information on offspring was collected at birth and at each required visit to child healthcare units (i.e., at 1, 3, 6, 9, 12, 18, 24, 30, and 36 months).

The socio-demographic and health-related characteristics of pregnant women were obtained from the prenatal health dataset from the EMRS. The information about the birth condition, postnatal feeding, and growth of the children were obtained from the birth registration information dataset and the conventional child health examination dataset from the EMRS. The study protocol was approved by the institutional review board of Zhejiang University School of Medicine (No. 2011-1-002).

### 2.2. Study Population

Participants who met the following criteria were included in the study: (1) maternal age ≥ 18 years old; (2) live singleton pregnancy; (3) gestational weeks of delivery between 37 and 42 weeks; (4) offspring had two or more health examination records (i.e., height and weight) between 12 and 36 months of age; (5) the feeding practices of offspring were recorded at each required visit before 12 months of age.

The exclusion criteria included: (1) maternal BMI in the first trimester <15 kg/m^2^ or >40 kg/m^2^; (2) offspring with congenital disease, chromosomal defects, or intrauterine infections; (3) abnormal data that could not be linked to the maternal prenatal health dataset, birth registration information, and conventional child health examination dataset.

### 2.3. Definition of Exposure Variables

In the process of data collection for this study, the feeding practices were categorized as full breastfeeding, partial breastfeeding, or formula feeding during the required health examination at 1, 3, 6, 9, and 12 months of age. According to the definition from the World Health Organization (WHO), breastfeeding practices are classified as exclusive, predominant, or partial breastfeeding [19,20]. Exclusive breastfeeding is defined as the provision of breast milk with no other liquids or solids, except for drops or liquid preparations containing vitamins, oral rehydration solutions, mineral supplements, or medications. Predominant breastfeeding is defined as the provision of breast milk, with the potential consumption of water or water-based drinks, fruit juice, oral rehydration salts, and drop or syrup forms of vitamins, minerals, and medicines. Partial breastfeeding is defined as infants fed with, but not limited to, breast milk and formula milk. In this study, the definition of full breastfeeding consists of exclusive and predominant breastfeeding [7]. Then, the duration of full breastfeeding was further categorized as <1 month, 1–2 months, 3–5 months and ≥6 months.

Pregnant women were required to receive a general screening for GDM with a 75 g oral glucose tolerance test (OGTT) at 24–28 weeks [21]. GDM was diagnosed by either a fasting glucose value ≥ 5.1 mmol/L, a 1 h post glucose value ≥ 10.0 mmol/L, or a 2 h post glucose value ≥ 8.5 mmol/L, according to the International Association for Diabetes in Pregnancy Study Groups (IADPSG) criteria [22].

Small for gestational age (SGA) infants, appropriate for gestational age (AGA) infants, and LGA infants were defined as those infants having an infant birth weight below the 10th percentile, between the 10th and 90th percentile, and above the 90th percentile of gestational age and gender-specific birth weights, respectively [23].

### 2.4. Definition of Overweight/Obesity

The measurements of weight (kg) and length (cm) were taken by specially trained research nurses at each child health examination. The BMI (kg/m^2^) was calculated as the weight divided by the length squared. According to the reference provided by the National Health Commission of the People’s Republic of China [24], children with BMI values greater than 1 standard deviation (SD) from the mean of sex- and age-specific BMI were classified as overweight/obese.

### 2.5. Covariates

The following variables were investigated as potential confounders: maternal age, maternal educational levels, early-pregnancy BMI, weight gain during pregnancy, parity, gravidity, hypertensive disorders of pregnancy, neonatal gender, gestational weeks at delivery, mode of delivery, and anthropometric measurements at 9 months of age.

### 2.6. Statistical Analysis

Continuous variables and categorical variables were expressed as the mean ± SD and frequencies (proportion), respectively. ANOVA tests and Pearson chi-square tests were applied to compare continuous variables and the distribution of categorical variables among multiple groups, respectively. Generalized estimating equation (GEE) models with an auto-regressive one-correlation structure were applied to analyze the associations of the duration of full breastfeeding with the risk of overweight/obesity in offspring aged between 12 and 36 months. The GEE model accounts for the correlation between repeated measurements of overweight over time within subjects and deals with missing values. The odds ratio (OR) and their 95% confidence intervals (CIs) for the association of full breastfeeding and overweight/obesity were reported as in [25]. Then, the above models were further adjusted for the covariates mentioned, and the adjusted ORs (aOR) with their 95% CIs were presented.

All analyses were performed using the statistical software R version 4.2.2. All statistical tests were two-sided, and a *p* value of less than 0.05 was considered statistically significant.

## 3. Results

### 3.1. Baseline Characteristics

Baseline characteristics of participants stratified by full breastfeeding duration are presented in Table 1. Among the total participants (*n* = 9329), 4368 (46.8%) were fully breastfed for <1 month, 783 (8.4%) for 1–2 months, 3318 (35.6%) for 3–5 months, and only 860 (9.2%) for ≥6 months. The mothers of infants who were full breastfeeding for <1 month were older and had a higher early-pregnancy BMI, along with a higher proportion of cesarean deliveries.

In comparison to infants of mothers without GDM (*n* = 7581), those of mothers with GDM (*n* = 1748) had a higher proportion of full breastfeeding for <1 month (46.0% vs. 50.4%) and a lower proportion of full breastfeeding for ≥6 months (9.3% vs. 8.8%) (Table 1). Similarly, compared with AGA infants (*n* = 7349), LGA infants (*n* = 1279) tended to have a shorter full-breastfeeding duration, although the difference was not statistically significant (Table 1).

### 3.2. Associations of the Duration of Full Breastfeeding with the Risk of Overweight/Obesity between the Ages of 12–36 Months

Associations between full breastfeeding duration and the risk of overweight/obesity for infants aged 12–36 months among all participants are presented in Table S1. In fully adjusted models, infants with full breastfeeding for 3–5 months (aOR: 0.75; 95% CI: 0.71, 0.80) and ≥6 months (aOR: 0.85; 95% CI: 0.77, 0.93) were positively associated with a reduced risk of overweight/obesity in early childhood compared with those with <1 month of full breastfeeding.

In this study, infants exposed to GDM showed a higher risk of overweight/obesity between the ages of 12–36 months compared with infants not exposed to GDM (aOR = 1.09; 95%CI: 1.01, 1.16). The associations of breastfeeding with the risk of overweight/obesity in early childhood stratified by GDM status are displayed in Table 2. Among infants whose mother did not have GDM, infants with full breastfeeding for 3–5 months (aOR: 0.87; 95% CI: 0.80, 0.94) and ≥6 months (aOR: 0.84; 95% CI: 0.74, 0.96) had a lower risk of overweight/obesity compared with those with <1 month of full breastfeeding. Among infants whose mother had GDM, infants with full breastfeeding for ≥6 months (aOR: 0.58; 95% CI: 0.44, 0.78) had a lower risk of overweight/obesity compared with those with <1 month of full breastfeeding. We further investigated the combined effects of breastfeeding and GDM status on early childhood overweight/obesity by a crossover analysis (Table S2). In comparison with infants with full breastfeeding for <1 month and whose mothers did not have GDM, those with full breastfeeding for ≥6 months and whose mothers had GDM had a decreased risk of overweight/obesity in early childhood (aOR: 0.63; 95% CI: 0.48, 0.83).

In this study, LGA infants had a higher risk of overweight/obesity between 12–36 months (aOR = 1.45; 95%CI: 1.35, 1.56) compared with AGA infants, while SGA infants had a lower risk (aOR = 0.51; 95%CI: 0.44, 0.59). The associations of breastfeeding with the risk of overweight/obesity in early childhood stratified by size at birth are presented in Table 3. AGA infants who received full breastfeeding for 3–5 months (aOR: 0.73; 95% CI: 0.68, 0.78) and ≥6 months (aOR: 0.84; 95% CI: 0.75, 0.93) showed a decreased risk of developing overweight/obesity compared with those who received <1 month of full breastfeeding. Similarly, LGA infants who received full breastfeeding for 3–5 months (aOR: 0.66; 95% CI: 0.57, 0.76) and ≥6 months (aOR: 0.70; 95% CI: 0.56, 0.88) showed a decreased risk of developing overweight/obesity compared with those with <1 month of full breastfeeding. We further investigated the combined effects of breastfeeding and size at birth on early childhood overweight/obesity by the crossover analysis (Table S3). In comparison with AGA infants with <1 month of full breastfeeding, LGA infants who received 3–5 months and ≥6 months of full breastfeeding were no longer at risk of overweight/obesity in early childhood.

Considering the combination effect of GDM and LGA (referring to LGA infants born to mothers with GDM), infants with full breastfeeding for 3–5 months (aOR: 0.62; 95% CI: 0.45, 0.85) and ≥6 months (aOR: 0.51; 95% CI: 0.31, 0.85) had a remarkably reduced risk of overweight/obesity compared with those with <1 month of full breastfeeding (Table S4).

## 4. Discussion

In this study, we found that increased breastmilk intake duration was associated with a reduced risk of overweight/obesity in early childhood. Meanwhile, our results indicated that the protective effect of breastfeeding against overweight/obesity in early childhood remained evident among LGA infants and infants born to mothers with GDM, and these high-risk infants experienced even greater benefits from a longer full breastfeeding duration.

Currently, with the advancements in formula milk products and intensified marketing efforts, breastfeeding faces unprecedented challenges in China [26,27,28,29]. Thus, emphasizing and evaluating the importance of breastfeeding is a pressing need. Consistent with previous studies [14,30,31,32], our finding confirmed the benefits of a longer exclusive/predominant breastfeeding duration in decreasing the risk of overweight/obesity in early childhood. These results support the recommendation from WHO’s global strategy that advocates exclusive breastfeeding for 6 months [20]. However, it is important to note that there is no consensus in the literature regarding the protective effects of breastfeeding against later obesity. Although some researchers attribute this inconsistency to residual confounding from variations in maternal sociodemographic characteristics [33], a recent meta-analysis including 159 studies suggested that breastfeeding reduced the odds of overweight/obesity, even after socioeconomic adjustments [34]. Thus, other external factors may contribute to this inconsistency. In addition, the results reported that male offspring tended to receive breastfeeding for a shorter duration compared with female offspring (deduced from the boy/girl ratio in each group). However, this phenomenon is not consistent in previous studies. For example, a study involving 57,201 children from seven provinces in China showed that boys appeared to receive a longer duration of breastfeeding [35], while another study suggested that girls might receive more breastfeeding [36]. Although we cannot determine the exact reasons for the longer duration of breastfeeding in the female offspring in this study, it may be because mothers perceive their sons as needing more nutrients, potentially leading to the earlier introduction of formula milk or other foods, as speculated by a nationwide study conducted in the United States [37].

Currently, the health consequences of breastfeeding by mothers with GDM remain controversial. One study found that longer breastfeeding was linked to increased weight/BMI gain from birth to 6 months in infants of mothers with GDM [14], while another study showed no impact on the early-life growth trajectory [13]. Similar to other studies [12,38], our findings suggest that breastfeeding for 6 months or longer significantly reduces the overweight/obesity risk in early childhood among infants of mothers with GDM, and the reduction in odds appears to be greater compared with infants of mothers without GDM. The inconsistent results may result from different degrees of glucose control and postpartum glycemia in GDM women across the various studies. Although data on postpartum glycemia levels are lacking in this study, our findings suggest that a longer breastfeeding duration is more likely to yield benefits than adverse effects in reducing the risk of overweight/obesity among infants born to mothers with GDM. Additionally, our study supports previous findings indicating that GDM influences the breastfeeding duration [39,40]. In our study, the proportion of GDM women who fully breastfed their infants for more than 3 months was significantly lower than that of women without GDM (41.7% vs. 45.6%). This disparity may be attributed to insulin resistance or inflammation in GDM women, which may diminish prolactin responsiveness and lead to delayed lactogenesis and breastfeeding initiation and maintenance [41,42].

LGA infants are at the greatest risk for future obesity, and research suggests that their postnatal growth in early childhood may be linked to long-term adverse outcomes [43,44]. Thus, promoting optimal postnatal growth is crucial for LGA infants. However, investigations focusing on the effects of breastfeeding, as a highly recommended approach to nutrition during the postnatal period, on early offspring overweight/obesity is limited. Our findings revealed that a longer duration of full breastfeeding was associated with a reduced risk of overweight/obesity in early childhood among AGA and LGA infants. This result confirmed that breastfeeding might be an appropriate way to achieve optimal postanal growth for LGA infants.

In fact, the potential mechanisms by which breastfeeding reduces the risk of childhood overweight/obesity are not fully elucidated. Extensive previous studies have suggested that certain bioactive components in breast milk, such as leptin, adiponectin, and insulin-like growth factor-1, may play important roles in reducing the risk of offspring overweight/obesity [45]. Therefore, when explaining the association between breastfeeding and early offspring weight gain among women with GDM, previous research also primarily focused on the impact of changes in the breast milk composition in mothers with GDM, such as the lower concentration of adiponectin [46,47]. However, epidemiological evidence on the association between GDM status and milk composition remains inconsistent. Our initial hypothesis was that perhaps the protective effect of breastfeeding against overweight/obesity may not remain in GDM pregnancies with suboptimal glycemic control. Due to the lack of data on late-pregnancy glycemic control in our study, the presence of LGA infants is considered indicative of more severe dysglycemia, as suggested by previous research [48]. However, we found that breastfeeding in infants born to mothers with GDM could still reduce the risk of overweight/obesity, even among those born LGA. This indirectly suggests that breastfeeding still has a protective effect against overweight/obesity in early childhood among poorly controlled GDM pregnancies. A recent prospective study innovatively found that the level of glucose in the breast milk of GDM mothers decreases compared with women without GDM, raising the speculation that milk glucose regulation may, at least partially, be independent of maternal blood glucose [49]. Furthermore, another study indicated that changes in breast milk composition mainly occur due to poor glycemic control during breastfeeding [50]. This implies that changes in breast milk composition in women with GDM may be temporary, with levels gradually returning to normal within a few weeks after delivery [51]. These findings may help to explain why increasing the duration of breastfeeding can still protect offspring born to mothers with GDM from the adverse effects of overweight/obesity.

In this study, we provide data from a large retrospective cohort with standardized procedures, and the findings underscore the importance of breastfeeding for early infant development. This is particularly crucial at a time when there is a perception that breastfeeding may not be necessary in modern urban settings. Meanwhile, considering that feeding practices are potentially modifiable, our findings could carry significant public health implications at a population level. In addition, we considered the effects of GDM and size at birth to further address the inconsistencies and missing data in the literature. However, some limitations still need to be noted. First, the report of feeding relied on recall at each required visit, and potential recall bias could have been introduced. Nevertheless, the recall of breastfeeding practices over a short period has been shown to be reliable [52]. Second, some potential confounders or variables, such as activity levels, time or sorts of solid food introduction, or frequency of breastfeeding were not available in this study. Meanwhile, we did not account for any treatments that women with GDM may have received, nor did we differentiate the degree of postpartum glucose control.

## 5. Conclusions

In conclusion, this study suggests that initiating and prolonging breastfeeding would reduce the risk of overweight/obesity in early childhood. Meanwhile, we observed that LGA infants and infants of mothers with GDM would experience greater benefits from a longer breastfeeding duration in terms of reducing the risk of overweight risk/obesity. These findings highlight the importance of promoting breastfeeding as an intervention against the epidemic of childhood overweight/obesity for high-risk populations. Further research should be conducted to investigate the underlying biological mechanisms.

## Figures and Tables

**Table 1:** Baseline characteristics of participants, stratified by the duration of full breastfeeding.

Characteristics	<1 Month	1–2 Months	3–5 Months	≥6 Months	*p*
N (%)	4368 (46.8)	783 (8.4)	3318 (35.6)	860 (9.2)	
Maternal age (years)	29.30 (4.09)	28.74 (4.06)	28.47 (3.72)	28.81 (3.70)	<0.001
Early-pregnancy BMI (kg/m^2^)	21.38 (3.09)	21.14 (3.02)	21.19 (2.76)	21.30 (2.87)	0.023
BMI category (*n*, %)					0.001
Underweight and normal	3660 (83.8)	674 (86.1)	2886 (87.0)	726 (84.4)	
Overweight and obese	708 (16.2)	109 (13.9)	432 (13.0)	134 (15.6)	
Gravidity (*n*, %)					0.007
1	1985 (45.4)	357 (45.6)	1587 (47.8)	399 (46.4)	
2	1149 (26.3)	193 (24.6)	881 (26.6)	218 (25.3)	
3	682 (15.6)	126 (16.1)	511 (15.4)	159 (18.5)	
≥4	552 (12.6)	107 (13.7)	339 (10.2)	84 (9.8)	
Parity (*n*, %)					<0.001
Primipara	2799 (64.1)	506 (64.6)	2217 (66.8)	543 (63.1)	
Multipara	1197 (27.4)	230 (29.4)	903 (27.2)	264 (30.7)	
Not known	372 (8.5)	47 (6.0)	198 (6.0)	53 (6.2)	
Maternal educational levels (*n*, %)					<0.001
Primary school or less	47 (1.1)	9 (1.1)	24 (0.7)	12 (1.4)	
Junior high school	582 (13.3)	117 (14.9)	354 (10.7)	112 (13.0)	
Senior high school	706 (16.2)	127 (16.2)	434 (13.1)	98 (11.4)	
College or higher	2275 (52.1)	403 (51.5)	1983 (59.8)	503 (58.5)	
Not known	758 (17.4)	127 (16.2)	523 (15.8)	135 (15.7)	
Weight gain during pregnancy (kg)	12.95 (4.01)	12.92 (3.65)	12.99 (3.71)	12.59 (3.85)	0.057
Gestational diabetes mellitus (*n*, %)					0.006
No	3487 (79.8)	635 (81.1)	2752 (82.9)	707 (82.2)	
Yes	881 (20.2)	148 (18.9)	566 (17.1)	153 (17.8)	
Hypertensive disorders of pregnancy (*n*, %)					0.058
No	4166 (95.4)	746 (95.3)	3199 (96.4)	831 (96.6)	
Hypertensive disorder	141 (3.2)	25 (3.2)	87 (2.6)	22 (2.6)	
Chronic hypertension	57 (1.3)	9 (1.1)	28 (0.8)	4 (0.5)	
Eclampsia	4 (0.1)	3 (0.4)	4 (0.1)	3 (0.3)	
Mode of delivery (*n*, %)					<0.001
Vaginal delivery	2399 (54.9)	445 (56.8)	2178 (65.6)	556 (64.7)	
Cesarean section	1969 (45.1)	338 (43.2)	1140 (34.4)	304 (35.3)	
Neonatal gender (*n*, %)					<0.001
Male	2413 (55.2)	407 (52.0)	1653 (49.8)	408 (47.4)	
Female	1955 (44.8)	376 (48.0)	1665 (50.2)	452 (52.6)	
Gestational age at delivery (years)	39.21 (1.14)	39.20 (1.16)	39.35 (1.10)	39.28 (1.12)	<0.001
Neonatal birth weight (g)	3358.09 (401.91)	3329.66 (422.85)	3365.27 (392.47)	3343.01 (404.22)	0.105
Size at birth (*n*, %)					0.174
SGA infant	332 (7.6)	69 (8.8)	230 (6.9)	70 (8.1)	
AGA infant	3405 (78.0)	606 (77.4)	2653 (80.0)	685 (79.7)	
LGA infant	631 (14.4)	108 (13.8)	435 (13.1)	105 (12.2)	

Abbreviation: AGA: appropriate for gestational age; BMI: body mass index; LGA: large for gestational age; SGA: small for gestational age.

**Table 2:** Associations between the duration of full breastfeeding and overweight/obesity in early childhood, stratified by GDM status.

Duration	N (%)	OR (95% CI)		aOR (95% CI)
Women without GDM				
<1 month	2914 (19.0)	Ref.		Ref.
1–2 months	518 (18.5)	0.96 (0.87, 1.07)		1.04 (0.92, 1.18)
3–5 months	1724 (14.2)	0.70 (0.66, 0.75)		0.87 (0.80, 0.94)
≥6 months	495 (15.9)	0.80 (0.72, 0.89)		0.84 (0.74, 0.96)
Women with GDM				
<1 month	788 (20.6)	Ref.		Ref.
1–2 months	125 (19.5)	0.94 (0.76, 1.16)		1.06 (0.82, 1.36)
3–5 months	335 (13.3)	0.59 (0.51, 0.68)		0.89 (0.75, 1.06)
≥6 months	105 (15.7)	0.72 (0.58, 0.90)		0.58 (0.44, 0.78)
P for interaction	0.034			

Abbreviation: aOR: adjusted odds ratio; CI: confidence interval; GDM: gestational diabetes mellitus; OR: odds ratio. Data adjusted for maternal age, maternal educational levels, parity, gravidity, hypertensive disorders of pregnancy, neonatal gender, gestational age at delivery, mode of delivery, early-pregnancy body mass index, weight gain during pregnancy, and related anthropometric measurements at 9 months.

**Table 3:** Associations between the duration of full breastfeeding and overweight/obesity in early childhood, stratified by size at birth.

Duration	N (%)	OR (95% CI)		aOR (95% CI)
SGA				
<1 month	103 (7.1)	Ref.		Ref.
1–2 months	22 (7.5)	1.07 (0.66, 1.73)		1.41 (0.78, 2.53)
3–5 months	65 (6.4)	0.90 (0.65, 1.24)		1.03 (0.70, 1.51)
≥6 months	25 (7.9)	1.12 (0.71, 1.76)		0.89 (0.46, 1.70)
AGA				
<1 month	2732 (18.3)	Ref.		Ref.
1–2 months	491 (18.3)	1.00 (0.89, 1.11)		1.02 (0.91, 1.13)
3–5 months	1539 (13.1)	0.67 (0.63, 0.72)		0.73 (0.68, 0.78)
≥6 months	452 (15.1)	0.79 (0.71, 0.88)		0.84 (0.75, 0.93)
LGA				
<1 month	867 (31.3)	Ref.		Ref.
1–2 months	130 (28.0)	0.86 (0.69, 1.06)		0.86 (0.69, 1.08)
3–5 months	455 (23.5)	0.67 (0.59, 0.77)		0.66 (0.57, 0.76)
≥6 months	123 (25.6)	0.75 (0.60, 0.94)		0.70 (0.56, 0.88)
P for interaction	<0.001			

Abbreviation: aOR: adjusted odds ratio; AGA: appropriate for gestational age; CI: confidence interval; LGA: large for gestational age; OR: odds ratio; SGA: small for gestational age. Data adjusted for maternal age, maternal educational levels, parity, gravidity, gestational diabetes mellitus status, hypertensive disorders of pregnancy, neonatal gender, gestational age at delivery, mode of delivery, early-pregnancy body mass index, weight gain during pregnancy, and related anthropometric measurements at 9 months.

## Data Availability

All datasets generated during and analyzed during the current study are not publicly available but are available from the corresponding author upon reasonable request.

## Supplementary Materials

The following supporting information can be downloaded at: https://www.mdpi.com/article/10.3390/nu16091351/s1, Table S1: Associations of the duration of full breastfeeding and the risk of overweight/obesity in early childhood among all participants. Table S2: Associations of GDM status and duration of full breastfeeding with the risk of overweight/obesity in early childhood. Table S3: Associations of size at birth and the duration of full breastfeeding with the risk of overweight/obesity in early childhood. Table S4: Associations of the duration of full breastfeeding with the risk of overweight/obesity in early childhood, stratified by GDM status during pregnancy and size at birth.

## References

[1] Afshin A., Forouzanfar M.H., Reitsma M.B., Sur P., Estep K., Lee A., Marczak L., Mokdad A.H., Moradi-Lakeh M., GBD Obesity Collaborators (2017). Health Effects of Overweight and Obesity in 195 Countries over 25 Years. N. Engl. J. Med..

[2] Pan X.F., Wang L., Pan A. (2021). Epidemiology and determinants of obesity in China. Lancet Diabetes Endocrinol..

[3] Panera N., Mandato C., Crudele A., Bertrando S., Vajro P., Alisi A. (2022). Genetics, epigenetics and transgenerational transmission of obesity in children. Front. Endocrinol..

[4] Consales A., Morniroli D., Vizzari G., Mosca F., Gianni M.L. (2022). Nutrition for Infant Feeding. Nutrients.

[5] Zheng M., D’Souza N.J., Atkins L., Ghobadi S., Laws R., Szymlek-Gay E.A., Grimes C., Baker P., He Q.Q., Campbell K.J. (2024). Breastfeeding and the Longitudinal Changes of Body Mass Index in Childhood and Adulthood: A Systematic Review. Adv. Nutr..

[6] Amaro A., Baptista F.I., Matafome P. (2022). Programming of future generations during breastfeeding: The intricate relation between metabolic and neurodevelopment disorders. Life Sci..

[7] Rzehak P., Oddy W.H., Mearin M.L., Grote V., Mori T.A., Szajewska H., Shamir R., Koletzko S., Weber M., Beilin L.J. (2017). Infant feeding and growth trajectory patterns in childhood and body composition in young adulthood. Am. J. Clin. Nutr..

[8] Oddy W.H., Mori T.A., Huang R.C., Marsh J.A., Pennell C.E., Chivers P.T., Hands B.P., Jacoby P., Rzehak P., Koletzko B.V. (2014). Early infant feeding and adiposity risk: From infancy to adulthood. Ann. Nutr. Metab..

[9] Trabulsi J.C., Smethers A.D., Eosso J.R., Papas M.A., Stallings V.A., Mennella J.A. (2020). Impact of early rapid weight gain on odds for overweight at one year differs between breastfed and formula-fed infants. Pediatr. Obes..

[10] Suwaydi M.A., Zhou X., Perrella S.L., Wlodek M.E., Lai C.T., Gridneva Z., Geddes D.T. (2022). The Impact of Gestational Diabetes Mellitus on Human Milk Metabolic Hormones: A Systematic Review. Nutrients.

[11] Hui L.L., Li A.M., Nelson E.A.S., Leung G.M., Lee S.L., Schooling C.M. (2018). In utero exposure to gestational diabetes and adiposity: Does breastfeeding make a difference?. Int. J. Obes..

[12] Gunderson E.P., Greenspan L.C., Faith M.S., Hurston S.R., Quesenberry C.P., SWIFT Offspring Study Investigators (2018). Breastfeeding and growth during infancy among offspring of mothers with gestational diabetes mellitus: A prospective cohort study. Pediatr. Obes..

[13] Dugas C., Kearney M., Perron J., Weisnagel S.J., Marc I., Robitaille J. (2021). Breastfeeding and growth trajectory from birth to 5 years among children exposed and unexposed to gestational diabetes mellitus in utero. J. Perinatol..

[14] Aris I.M., Soh S.E., Tint M.T., Saw S.M., Rajadurai V.S., Godfrey K.M., Gluckman P.D., Yap F., Chong Y.S., Lee Y.S. (2017). Associations of infant milk feed type on early postnatal growth of offspring exposed and unexposed to gestational diabetes in utero. Eur. J. Nutr..

[15] Patro Golab B., Santos S., Voerman E., Lawlor D.A., Jaddoe V.W.V., Gaillard R., MOCO Study Group Authors (2018). Influence of maternal obesity on the association between common pregnancy complications and risk of childhood obesity: An individual participant data meta-analysis. Lancet Child Adolesc. Health.

[16] Scifres C.M. (2021). Short- and Long-Term Outcomes Associated with Large for Gestational Age Birth Weight. Obstet. Gynecol. Clin. N. Am..

[17] Jiang W., Mo M., Si S., Wu J., Pu L., Huang M., Shao B., Xin X., Wang S., Shen Y. (2022). Association of hypertensive disorders of pregnancy with infant growth in the first 36 months of life. Eur. J. Pediatr..

[18] Peng Z., Si S., Cheng H., Zhou H., Chi P., Mo M., Zhuang Y., Liu H., Yu Y. (2022). The Associations of Maternal Hemoglobin Concentration in Different Time Points and Its Changes during Pregnancy with Birth Weight Outcomes. Nutrients.

[19] World Health Organization (2008). Indicators for Assessing Infant and Young Child Feeding Practices.

[20] World Health Organization (2003). Global Strategy for Infant and Young Child Feeding.

[21] Subgroup, Obstetrics, and Chinese Medical Association Gynecology (2018). Guideline of preconception and prenatal care (2018). Zhonghua Fu Chan Ke Za Zhi.

[22] Metzger B.E., Gabbe S.G., Persson B., Lowe L.P., Dyer A.R., Oats J.J.N., Buchanan T.A. (2010). International association of diabetes and pregnancy study groups recommendations on the diagnosis and classification of hyperglycemia in pregnancy. Diabetes Care.

[23] National Health Commission of the People’s Republic of China (2022). Growth Standard for Newborns by Gestational Age.

[24] Growth Standard for Children under 7 Years of Age. http://www.nhc.gov.cn/wjw/fyjk/202211/16d8b049fdf547978a910911c19bf389.shtml.

[25] Pluymen L.P.M., Wijga A.H., Gehring U., Koppelman G.H., Smit H.A., van Rossem L. (2018). Early introduction of complementary foods and childhood overweight in breastfed and formula-fed infants in the Netherlands: The PIAMA birth cohort study. Eur. J. Nutr..

[26] Ndirangu M.N., Gatimu S.M., Mwinyi H.M., Kibiwott D.C. (2018). Trends and factors associated with early initiation of breastfeeding in Namibia: Analysis of the Demographic and Health Surveys 2000–2013. BMC Pregnancy Childbirth.

[27] Wallenborn J.T., Levine G.A., Carreira Dos Santos A., Grisi S., Brentani A., Fink G. (2021). Breastfeeding, Physical Growth, and Cognitive Development. Pediatrics.

[28] Zhou Q., Feng X.L. (2023). Breastfeeding practices in Northeast China in 2008 and 2018: Cross-sectional surveys to explore determinants over a decade. Int. Breastfeed. J..

[29] Duan Y., Yang Z., Lai J., Yu D., Chang S., Pang X., Jiang S., Zhang H., Bi Y., Wang J. (2018). Exclusive Breastfeeding Rate and Complementary Feeding Indicators in China: A National Representative Survey in 2013. Nutrients.

[30] Kramer M.S., Guo T., Platt R.W., Vanilovich I., Sevkovskaya Z., Dzikovich I., Michaelsen K.F., Dewey K. (2004). Feeding effects on growth during infancy. J. Pediatr..

[31] Griffiths L.J., Smeeth L., Hawkins S.S., Cole T.J., Dezateux C. (2009). Effects of infant feeding practice on weight gain from birth to 3 years. Arch. Dis. Child.

[32] Gunnarsdottir I., Schack-Nielsen L., Michaelsen K.F., Sorensen T.I., Thorsdottir I. (2010). Infant weight gain, duration of exclusive breast-feeding and childhood BMI—Two similar follow-up cohorts. Public Health Nutr..

[33] Fall C.H.D., Kumaran K. (2019). Metabolic programming in early life in humans. Philos. Trans. R. Soc. B Biol. Sci..

[34] Horta B.L., Rollins N., Dias M.S., Garcez V., Perez-Escamilla R. (2023). Systematic review and meta-analysis of breastfeeding and later overweight or obesity expands on previous study for World Health Organization. Acta Paediatr..

[35] Liu J., Gao D., Li Y., Chen M., Wang X., Ma Q., Ma T., Chen L., Ma Y., Zhang Y. (2022). Breastfeeding Duration and High Blood Pressure in Children and Adolescents: Results from a Cross-Sectional Study of Seven Provinces in China. Nutrients.

[36] Cheng T.S., Kwok M.K., Leung G.M., Schooling C.M. (2018). The Associations of Breast Feeding with Infant Growth and Body Mass Index to 16 years: ‘Children of 1997’. Paediatr. Perinat. Epidemiol..

[37] Shafer E.F., Hawkins S.S. (2017). The Impact of Sex of Child on Breastfeeding in the United States. Matern. Child Health J..

[38] Schaefer-Graf U.M., Hartmann R., Pawliczak J., Passow D., Abou-Dakn M., Vetter K., Kordonouri O. (2006). Association of breast-feeding and early childhood overweight in children from mothers with gestational diabetes mellitus. Diabetes Care.

[39] Loewenberg Weisband Y., Rausch J., Kachoria R., Gunderson E.P., Oza-Frank R. (2017). Hospital Supplementation Differentially Impacts the Association Between Breastfeeding Intention and Duration Among Women With and Without Gestational Diabetes Mellitus History. Breastfeed. Med..

[40] Oza-Frank R., Moreland J.J., McNamara K., Geraghty S.R., Keim S.A. (2016). Early Lactation and Infant Feeding Practices Differ by Maternal Gestational Diabetes History. J. Hum. Lact..

[41] Matias S.L., Dewey K.G., Quesenberry C.P., Gunderson E.P. (2014). Maternal prepregnancy obesity and insulin treatment during pregnancy are independently associated with delayed lactogenesis in women with recent gestational diabetes mellitus. Am. J. Clin. Nutr..

[42] Rasmussen K.M., Kjolhede C.L. (2004). Prepregnant overweight and obesity diminish the prolactin response to suckling in the first week postpartum. Pediatrics.

[43] Lei X., Zhao D., Huang L., Luo Z., Zhang J., Yu X., Zhang Y. (2018). Childhood Health Outcomes in Term, Large-for-Gestational-Age Babies With Different Postnatal Growth Patterns. Am. J. Epidemiol..

[44] Viswanathan S., McNelis K., Makker K., Calhoun D., Woo J.G., Balagopal B. (2022). Childhood obesity and adverse cardiometabolic risk in large for gestational age infants and potential early preventive strategies: A narrative review. Pediatr. Res..

[45] Martin C.R., Ling P.R., Blackburn G.L. (2016). Review of Infant Feeding: Key Features of Breast Milk and Infant Formula. Nutrients.

[46] Aydin S. (2010). The presence of the peptides apelin, ghrelin and nesfatin-1 in the human breast milk, and the lowering of their levels in patients with gestational diabetes mellitus. Peptides.

[47] Yu X., Rong S.S., Sun X., Ding G., Wan W., Zou L., Wu S., Li M., Wang D. (2018). Associations of breast milk adiponectin, leptin, insulin and ghrelin with maternal characteristics and early infant growth: A longitudinal study. Br. J. Nutr..

[48] Kaul P., Bowker S.L., Savu A., Yeung R.O., Donovan L.E., Ryan E.A. (2019). Association between maternal diabetes, being large for gestational age and breast-feeding on being overweight or obese in childhood. Diabetologia.

[49] Choi Y., Nagel E.M., Kharoud H., Johnson K.E., Gallagher T., Duncan K., Kharbanda E.O., Fields D.A., Gale C.A., Jacobs K. (2022). Gestational Diabetes Mellitus Is Associated with Differences in Human Milk Hormone and Cytokine Concentrations in a Fully Breastfeeding United States Cohort. Nutrients.

[50] Dugas C., Perron J., Kearney M., Mercier R., Tchernof A., Marc I., Weisnagel S.J., Robitaille J. (2017). Postnatal Prevention of Childhood Obesity in Offspring Prenatally Exposed to Gestational Diabetes mellitus: Where Are We Now?. Obes. Facts.

[51] Aydin S., Geckil H., Karatas F., Donder E., Kumru S., Kavak E.C., Colak R., Ozkan Y., Sahin I. (2007). Milk and blood ghrelin level in diabetics. Nutrition.

[52] Li R., Scanlon K.S., Serdula M.K. (2005). The validity and reliability of maternal recall of breastfeeding practice. Nutr. Rev..

