# Supplementary material for: Breastfeeding, Gestational Diabetes Mellitus, Size at Birth and Overweight/Obesity in Early Childhood
Source: Nutrients. 2024 Apr 30;16(9):1351. doi: 10.3390/nu16091351 (PMC11085597; doi:10.3390/nu16091351)
Supplement: Supplementary file 1 [file nutrients-16-01351-s001.zip › nutrients-2975380-supplementary.pdf]

Table S1. Associations of duration of full breastfeeding and the risk of overweight/obesity in early childhood among all participants.

Duration	N (%)	OR (95% CI)	aOR (95% CI)
<1 month	3702 (19.3)	Ref.	Ref.
1-2 months	643 (18.7)	**0.96 (0.87, 1.05)**	0.99 (0.90, 1.09)
3-5 months	2059 (14.0)	**0.68 (0.64, 0.72)**	**0.75 (0.71, 0.80)**
≥ 6 months	600 (15.8)	**0.78 (0.71, 0.86)**	**0.85 (0.77, 0.93)**

Abbreviation: aOR: adjusted odds ratio; CI: confidence intervals; OR: odds ratio

Adjusted for: maternal age, maternal educational levels, parity, gravidity, gestational diabetes mellitus status, hypertensive disorders of pregnancy, neonatal gender, gestational age at delivery, mode of delivery, early-pregnancy body mass index, weight gain during pregnancy, anthropometric measurements at 9 months.

Table S2. Associations of GDM status and duration of full breastfeeding with the risk of overweight/obesity in early childhood.

GDM	Duration	OR (95% CI)	aOR (95% CI)
No	<1 month	Ref.	Ref.
No	1-2 months	**0.96 (0.87, 1.07)**	1.04 (0.92, 1.18)
No	3-5 months	**0.70 (0.66, 0.75)**	**0.87 (0.80, 0.94)**
No	≥ 6 months	**0.80 (0.72, 0.89)**	**0.83 (0.73, 0.95)**
Yes	<1 month	**1.10 (1.01, 1.20)**	**1.11 (1.02, 1.21)**
Yes	1-2 months	1.03 (0.85, 1.26)	1.10 (0.87, 1.40)
Yes	3-5 months	**0.65 (0.57, 0.73)**	0.97 (0.84, 1.12)
Yes	≥ 6 months	**0.79 (0.64, 0.98)**	**0.63 (0.48, 0.83)**

Abbreviation: aOR: adjusted odds ratio; CI: confidence intervals; GDM: gestational diabetes mellitus; OR: odds ratio

Adjusted for: maternal age, maternal educational levels, parity, gravidity, hypertensive disorders of pregnancy, neonatal gender, gestational age at delivery, mode of delivery, early-pregnancy body mass index, weight gain during pregnancy, anthropometric measurements at 9 months

Table S3. Associations of size at birth and duration of full breastfeeding with the risk of overweight/obesity in early childhood.

Size at birth	Duration	OR (95% CI)	aOR (95% CI)
SGA	<1 month	**0.34 (0.28, 0.42)**	**0.41 (0.33, 0.50)**
SGA	1-2 months	**0.36 (0.23, 0.56)**	**0.44 (0.28, 0.68)**
SGA	3-5 months	**0.30 (0.24, 0.39)**	**0.42 (0.33, 0.55)**
SGA	≥ 6 months	**0.38 (0.25, 0.57)**	**0.55 (0.36, 0.83)**
AGA	<1 month	Ref.	Ref.
AGA	1-2 months	1.00 (0.89, 1.11)	1.01 (0.91, 1.13)
AGA	3-5 months	**0.67 (0.63, 0.72)**	**0.72 (0.67, 0.77)**
AGA	≥ 6 months	**0.79 (0.71, 0.88)**	**0.83 (0.74, 0.92)**
LGA	<1 month	**2.03 (1.86, 2.23)**	**1.55 (1.41, 1.71)**
LGA	1-2 months	**1.74 (1.41, 2.14)**	**1.41 (1.14, 1.73)**
LGA	3-5 months	**1.37 (1.22, 1.54)**	1.12 (0.99, 1.25)
LGA	≥ 6 months	**1.53 (1.24, 1.89)**	1.17 (0.94, 1.45)

Abbreviation: aOR: adjusted odds ratio; AGA: appropriate for gestational age; CI: confidence intervals; LGA: large for gestational age; OR: odds ratio; SGA: small for gestational age

Adjusted for: maternal age, maternal educational levels, parity, gravidity, gestational diabetes mellitus status, hypertensive disorders of pregnancy, neonatal gender, gestational age at delivery, mode of delivery, early-pregnancy body mass index, weight gain during pregnancy, anthropometric measurements at 9 months

Table S4. Associations of duration of full breastfeeding with the risk of overweight/obesity in early childhood, stratified by GDM status during pregnancy and size at birth.

Duration	N (%)	OR (95% CI)	aOR (95% CI)
**Without GDM & SGA**			
<1 month	74 (6.3)	Ref.	Ref.
1-2 months	20 (7.6)	1.23 (0.73, 2.05)	1.56 (0.83, 2.92)
3-5 months	62 (7.1)	1.13 (0.80, 1.60)	1.11 (0.72, 1.72)
≥ 6 months	18 (6.6)	1.04 (0.61, 1.77)	0.87 (0.45, 1.70)
**Without GDM & AGA**			
<1 month	2200 (18.2)	Ref.	Ref.
1-2 months	406 (18.4)	1.01 (0.90, 1.14)	1.06 (0.94, 1.20)
3-5 months	1294 (13.3)	**0.69 (0.64, 0.74)**	**0.75 (0.70, 0.81)**
≥ 6 months	377 (15.2)	**0.81 (0.72, 0.91)**	**0.86 (0.77, 0.98)**
**Without GDM & LGA**			
<1 month	640 (31.0)	Ref.	Ref.
1-2 months	92 (26.7)	0.81 (0.63, 1.05)	0.80 (0.61, 1.04)
3-5 months	368 (23.8)	**0.69 (0.60, 0.81)**	**0.66 (0.56, 0.77)**
≥ 6 months	100 (26.9)	**0.81 (0.64, 1.04)**	**0.75 (0.58, 0.97)**
**GDM & SGA**			
<1 month	29 (10.0)	Ref.	Ref.
1-2 months	2 (6.2)	0.63 (0.14, 2.82)	0.29 (0.03, 3.12)
3-5 months	3 (2.1)	**0.19 (0.06, 0.64)**	**0.19 (0.04, 0.79)**
≥ 6 months	7 (15.9)	1.70 (0.68, 4.23)	1.79 (0.13, 24.60)
**GDM & AGA**			
<1 month	532 (18.8)	Ref.	Ref.
1-2 months	85 (17.4)	0.92 (0.71, 1.18)	0.88 (0.68, 1.14)
3-5 months	245 (12.3)	**0.60 (0.51, 0.71)**	**0.61 (0.51, 0.72)**
≥ 6 months	75 (14.6)	**0.73 (0.56, 0.95)**	**0.69 (0.52, 0.91)**
**GDM & LGA**			
<1 month	227 (32.1)	Ref.	Ref.
1-2 months	38 (31.7)	0.99 (0.65, 1.50)	1.00 (0.65, 1.52)
3-5 months	87 (22.2)	**0.61 (0.45, 0.81)**	**0.62 (0.45, 0.85)**
≥ 6 months	23 (21.1)	**0.57 (0.35, 0.92)**	**0.51 (0.31, 0.85)**

Abbreviation: aOR: adjusted odds ratio; AGA: appropriate for gestational age; CI: confidence intervals; GDM: gestational diabetes mellitus; LGA: large for gestational age; OR: odds ratio; SGA: small for gestational age. **Adjusted for:** maternal age, maternal educational levels, parity, gravidity, hypertensive disorders of pregnancy, neonatal gender, gestational age at delivery, mode of delivery, early-pregnancy body mass index, weight gain during pregnancy, anthropometric measurements at 9 months
